# Sero-Epidemiological Survey of Human Cystic Echinococcosis in Kashmir, North India

**DOI:** 10.1371/journal.pone.0124813

**Published:** 2015-04-27

**Authors:** Bashir Ahmad Fomda, Asiya Khan, Manzoor Ahmad Thokar, Ajaz Ahmad Malik, Anjum Fazili, Rayees Ahmad Dar, Monika Sharma, Nancy Malla

**Affiliations:** 1 Department of Microbiology, Sher-i-Kashmir Institute of Medical Sciences, Srinagar, Jammu and Kashmir, India; 2 Department of General Surgery, Sher-i-Kashmir Institute of Medical Sciences, Srinagar, Jammu and Kashmir, India; 3 Department of Community Medicine, Sher-i-Kashmir Institute of Medical Sciences, Srinagar, Jammu and Kashmir, India; 4 Department of Biostatistics, Sher-i-Kashmir Institute of Medical Sciences, Srinagar, Jammu and Kashmir, India; 5 Department of Medical Parasitology, Post Graduate Institute of Medical Education and Research, Chandigarh, India; Aga Khan University Hospital Nairobi, KENYA

## Abstract

**Background:**

Echinococcosis is a human and animal health problem in many endemic areas worldwide. There are numerous reports and hospital-based studies from Kashmir, North India, yet there has been no epidemiological study conducted in Kashmir, the apparently endemic area for human hydatidosis. This study was designed to determine the seroprevalence of hydatid infection in Kashmir Valley and to find out association of risk factors for acquisition of this infection.

**Methodology:**

Fourteen hundred and twenty-nine samples were collected from different districts in the Kashmir region (North India) using systematic random sampling. The 130 control samples included were from apparently healthy blood donors (100), patients with other parasitic infections (20), surgically confirmed hydatidosis patients (5), and apparently healthy subjects excluded for hydatidosis and intestinal parasitic infections (5). Hydatid-specific IgG antibody was detected by enzyme-linked immunosorbent assay, and seropositive samples were analysed further by Western blotting.

**Results:**

Out of 1,429 samples, 72 (5.03%) were IgG positive by ELISA. The percentage occurrence of the highly immunoreactive antigenic fractions in IgG ELISA positive samples was 57 kDa (72.2%) followed by 70 kDa (66.7%) and 39kDa (58.3%) by immunoblotting. Samples with other parasitic infections were reactive with the cluster of 54-59 kDa antigenic fractions. Age <15 years, male gender, contact with dog, and rural residence were the most significant factors associated with the seropositivity.

**Conclusion:**

The study revealed that 72 (5.03%) out of 1,429 subjects asymptomatic for hydatidosis were seropositve to *E*.*granulosus* antigen by ELISA. Western blot analysis of 72 ELISA seropositive samples showed that 66.7% and 58.3% of samples were immunoreactive with 70 and 39kDa specific antigenic fractions, respectively. The seropositivity was significantly higher (5.79%) in the younger age group (<15 years) as compared to the 16-55 years (4.07%) and > 55 years (3.05%) age groups, suggesting ongoing transmission of this infection in the younger age group. The number of seropositive males was significantly higher as compared to females. The risk factors identified were rural residence and contact with dogs. The study suggests the presence of asymptomatic infection in subjects in Kashmir, North India, and efforts need to be made for implementation of effective prevention measures to reduce the infection burden, which may otherwise lead to symptomatology and complications in the infected subjects.

## Introduction

Cystic echinococcosis or hydatidosis, caused by the larval stage of *Echinococcus granulosus*, is considered one of the most important zoonotic helminthic diseases throughout the world. *E*. *granulosus* is a complex of species/strains which exhibit diversity in their life cycle patterns and host range. Globally the burden of disease is very high and causes dramatic changes in terms of human and veterinary affairs. The World Health Organization has recently included echinococcosis in its strategic plans for the control of neglected tropical diseases. It is common in sheep farming regions like Australia, New Zealand, China, South America, Middle East, African countries around the Mediterranean and in India [1–7].

The main source of income in the majority of rural population in Kashmir Valley, Jammu and Kashmir State in North India is agriculture and livestock grazing (sheep and cattle). Moreover, there is a huge population of stray dogs. The slaughtering of livestock without veterinary control, the widespread rural practice of feeding dogs with the viscera of home butchered sheep is a common practice. All these factors are highly favorable for transmission of echinococcosis.

There are numerous reports and hospital based studies from Srinagar Kashmir [3, 7–16], yet there is no epidemiological study reported from Kashmir, the apparently endemic area for human hydatidosis. Knowing the burden of disease is highly important, so that the necessary measures for eradication and/ or control of the disease can be adapted. The present study was designed to determine the seroprevalence of human hydatid infection by ELISA using hydatid cyst fluid antigen, identification of immunoreactive antigens in IgG seropositive samples by Western blotting and to find out association of risk factors for acquisition of this infection in Kashmiri population.

## Materials and Methods

### Study area and population

**Fig 1:**
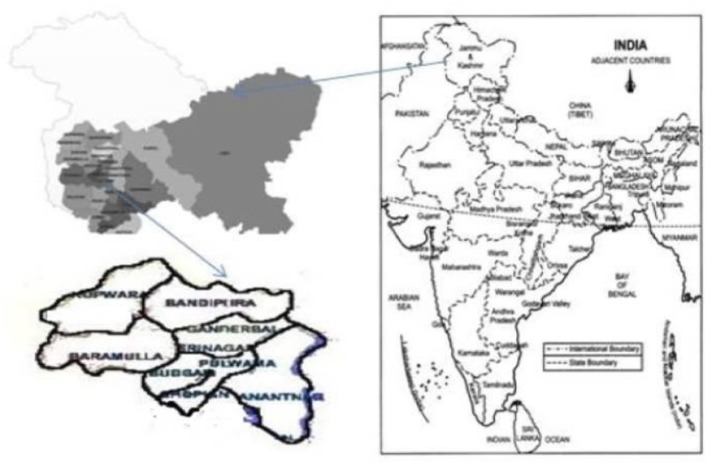
The geographic location of the surveyed population in Jammu and Kashmir, North India. Right: Map of India, Left up: Kashmir Valley within State of Jammu and Kashmir and Left down: Map of Kashmir Valley showing nine districts where study was conducted.

Jammu & Kashmir State constitutes the northern most extremity of India. It is situated between 32 degree and 37 degree north latitude and 73 degree and 80 degree east longitude. The projected population of the state is 12.55 million. The State with its summer and winter capitals at Srinagar and Jammu respectively is divided into three regions: Kashmir Valley, Jammu, and Ladakh. Kashmir Valley has 10 districts having a population of 5.35 million. The study population was selected randomly from 23 villages of nine districts in the Kashmir Valley using systematic random sampling (Fig 1). All the subjects in the households irrespective of age and gender were screened. Consent was obtained from subjects prior to enrollment in the study and in case of minor consent was obtained from parents.

Subjects enrolled in the study comprised of 1429 asymptomatic individuals residing in nine districts of Kashmir Valley. A detailed demographic characteristics and relevant history was recorded (e.g. age, gender, place of residence, contact with dogs etc). In addition, apparently healthy blood donors (100), who attended Sher-i-Kashmir Institute of Medical Sciences (SKIMS) for blood donation were included to find out baseline titer and to calculate cut-off OD (Optical density). Five patients found positive for ascariasis by stool examination and 15 samples seropositive for amoebiasis, toxoplasmosis and neurocysticercosis (five each) collected at PGIMER, Chandigarh were included to assess the cross reactivity. Five surgically confirmed hydatidosis patients were included as positive controls and five apparently healthy individuals, mainly staff members (excluded for hydatid infection by specific antibody detection by ELISA and intestinal parasitic infections by stool examination) served as negative controls.

Five milliliter of blood sample was withdrawn from each study subject and control after their due consent. Serum was separated and divided into two aliquots one each for ELISA and Western blotting. The seropositive samples were transported under refrigerated conditions to Post Graduate Institute of Medical Education and Research (PGIMER), Chandigarh, India for further analysis by Western blotting.

### Hydatid specific IgG antibody detection by ELISA

Hydatid cyst was obtained from sheep slaughtered at a local abattoir. Hydatid fluid was aspirated aseptically from fertile hydatid cyst and centrifuged at 2000xg for 45 min. The fluid was passed through Whatman WCN type membrane filter (cellulose nitrate, 47 mm diameter, 0.45 μm pore size) and dialysed against distilled water overnight at 4°C using dialysis tubing (Sigma, USA) with molecular weight cut-off 2000 Da [7,16]. The antigen protein concentration was estimated by the method of Lowry *et al*., with bovine serum albumin (BSA) as a reference standard [17]. The protein concentration of antigen was 1.3mg/ml.

Hydatid specific IgG antibody detection in serum was performed by indirect enzyme linked immunosorbent assay (ELISA) as detailed earlier [7, 16]. The optimum concentration of the antigen and goat anti-human horseradish peroxidase-conjugated IgG (Sigma, USA) was determined by the checker board titration method and was found to be 2 μg per well and 1:4000 respectively.

Samples from 100 apparently healthy blood donors along with five surgically confirmed patients (positive control) and five apparently healthy individuals, excluded for intestinal parasitic infections (negative control) were initially tested in doubling dilutions (1:40 to 1:2560) by ELISA to arrive at a cut-off serum dilution [18]. A dilution of 1:640 was used as cut-off titer.

For detection of antibody response in test samples (1429), briefly, 96 well microtitre plates were coated with 2μg of antigen /100μl/ well diluted in 0.1M carbonate/bicarbonate buffer (pH 9.6). All the test and control sera were diluted 1:640 and 100 μl was added to each well of microtiter plates. The plates were incubated for one hour at 37°C and then washed three times with PBS-Tween and goat anti-human horse radish peroxidase-conjugated IgG diluted in PBS-Tween was added to each well. The plates were incubated for one hour at 37°C and were then washed three times with PBS-Tween. A substrate solution of 3',3',5',5' tetramethylbenzedine (TMB) was added and incubated at room temperature for 15–20 minutes. Color development was stopped with 2M sulfuric acid (100 μl) and absorbance values (ODs) were measured at 450 nm with an ELISA plate reader (Bio Rad, USA).

The test samples (1429) were tested in separate batches and five positive controls, five negative control samples along with substrate and buffer blanks were included in each plate to avoid plate to plate variation [7]. All seropositve patients were referred to the Department of General surgery, SKIMS, Srinagar for further investigations and management as required.

### Sodium dodecyl sulphate polyacrylamide gel electrophoresis (SDS-PAGE) and Western blot assay

Hydatid fluid antigen was separated on 12.8% separating gel and 4% stacking gel as described previously with slight modifications [19]. The optimum concentration (450μg/ 10x10 cm gel) of hydatid fluid antigen under reducing conditions in the presence of β-mercaptoethanol was added in the reference well. A pre stained standard protein ladder marker (thermo scientific) was also run. Electrophoresis was run at constant voltage (130 volts) for one hour in Mini Bio-Rad apparatus. Antigenic fractions in the gel were transferred (blotted) onto the nitrocellulose membrane by using the “Locking gel cassette clamping system” (Bio-Rad apparatus).

Nitrocellulose strips containing the antigen were blocked by 3% BSA in PBS-Tween 20, overnight at 4°C followed by five washings with PBS-T20 buffer. The washed strips were incubated with serum samples (predetermined optimum dilution 1: 400) for 2.5 hours at room temperature under constant rocking. Unbound components were removed by five washings with PBS-T and strips were incubated at room temperature with optimum dilution (1:2000) of goat anti-human horse radish peroxidase-conjugated IgG (Sigma-Aldrich) for 2.5 hours. The strips were washed again, placed in DAB (3ʹ, 3ʹ-diaminobenzidine) and hydrogen peroxide substrate solution and the results were interpreted.

Reactive immunogenic fractions were noted in the Gel Doc System (UVI pro) and the results were interpreted by software UVI Photo MW v 1.0 (Cambridge U.K). Serum samples reactive with one or more antigenic fraction were considered positive. Frequency (percentage) of each reactive fraction was calculated by counting the occurrence of reactive band in total number of reactive samples/total number of reactive samples x 100 [20].

### Ethical clearance

The study was approved by the ethical clearance committees of Sher-i-Kashmir Institute of Medical Sciences Srinagar, Kashmir and Post Graduate Institute of Medical Education and Research, Chandigarh, India. Consent was obtained from subjects prior to enrolment in the study and in case of minor consent was obtained from parents.

### Data analysis

Data was analysed using the statistical package SPSS (v. 20). Chi square test was used for qualitative data in univariate analysis to find out association of various significant risk factors and binary logistic regression (multivariate) analysis was employed to eliminate confounding factors significantly associated with seropositivity. The results were considered statistically significant when the P value was ≤0.05.

## Results

### Demographic characteristics and IgG antibody positivity by ELISA

Fourteen hundred twenty-nine subjects included in this study were grouped in the age range of <15 years (148), 16–55 years (1015) and > 55 years (266). Out of them 630 were males and 799 were females. Out of 1429 samples, 72 (5.03%) were found positive for IgG antibodies by ELISA. The demographic characteristics of the studied population in terms of gender, place of residence and age group are presented in Table 1. High prevalence was observed in district Anantnag (9.23%) followed by district Baramulla (8.5%), Bandipora (8.0%), Kupwara (3.48%), Srinagar (3.28%), and Budgam (1.82%). None of the samples were positive in district Ganderbal, Shopian and Pulwama (Table 1).

**Table 1:** District and village wise distribution of demographic characteristics and number of seropositive subjects (N = 1429).

District	Village (Urban or Rural)	No. of subjects studied	Male	Female	Age (Seropositive/Total)			Seropositive/ Total Subjects (%)	P-value
					1–15 Years	16–55 Years	>55 Years		
1. Baramulla	Kripalpora (Rural)	96	48	48	0/12	08/78	0/6	08/96 (8.33)	
	Wadoora (Rural)	47	14	33	0/4	01/11	02/32	03/47 (6.38)	
	Nowpora (Rural)	55	22	33	04/12	02/33	0/10	06/55 (10.91)	
	**Total**	**198**	**84**	**114**	**04/28**	**11/122**	**02/48**	**17/198 (8.5)**	**0.128**
2. Anantnag	Lalan (Rural)	167	68	99	05/20	13/115	05/32	23/167(13.77)	
	Bijbehara (Urban)	46	26	20	0	0/34	0/12	0/46(0)	
	Anzwalla (Urban)	36	10	26	0/28	0/8	0	0/36(0)	
	**Total**	**249**	**104**	**145**	**05/48**	**13/157**	**05/44**	**23/249 (9.23)**	
3. Bandipora	Nowgam (Rural)	172	90	82	05/19	08/123	01/30	14/172 (8.14)	
	Asham (Rural)	103	55	48	04/13	02/72	02/18	08/103 (7.77)	
	**Total**	**275**	**145**	**130**	**09/32**	**10/195**	**03/48**	**22/275 (8.0)**	**0.10**
4. Kupwara	Pazipoora (Urban)	44	14	30	0/5	0/29	0/10	0/44(0)	
	Chogul (Rural)	42	26	16	01/10	02/30	0/2	03/42 (7.14)	
	**Total**	**86**	**40**	**46**	**01/15**	**02/59**	**0/12**	**03/86 (3.48)**	**0.04**
5. Budgam	Sozeith (Rural)	41	26	15	0/8	01/27	01/6	02/41 (4.88)	
	Kawoosa (Urban)	32	12	20	0/2	02/23	0/7	02/32 (6.25)	
	Mazhoma (Urban)	49	29	20	0/3	0/37	0/9	0/46(0)	
	Manglar (Rural)	46	6	40	0/2	0/37	01/7	01/46 (2.17)	
	Kralwari (Rural)	106	38	68	0/2	0/93	0/11	0/106(0)	
	**Total**	**274**	**111**	**163**	**0/17**	**03/217**	**02/40**	**05/274 (1.82)**	**0.0001**
6. Ganderbal	Tulbagh (Rural)	13	8	5	0/2	0/9	0/2	0/13	
	Wusun (Rural)	20	7	13	0/1	0/14	0/5	0/20	
	**Total**	**33**	**15**	**18**	**0/3**	**0/23**	**0/7**	**0/33**	**0.04**
7. Shopian	Babapora (Rural)	47	14	33	0/3	0/41	0/3	0/47	
	Safanagri (Rural)	66	28	38	0	0/50	0/16	0/66	
	Wachi (Rural)	76	29	47	0/1	0/65	0/10	0/76	
	**Total**	**189**	**71**	**118**	**0/4**	**0/156**	**0/29**	**0/189(0)**	**0**
8. Pulwama	Ratsun (Rural)	**64**	**34**	**30**	**0/1**	**0/37**	**0/26**	**0/64(0)**	**0.004**
9. Srinagar	Srinagar (Urban)	**61**	**26**	**35**	**0**	**02/49**	**0/12**	**02/61 (3.28)**	**0.06**
	**Total**	**1429**	**630**	**799**	**19/148**	**41/1015**	**12/266**	**72/1429 (5.03)**	

**Fig 2:**
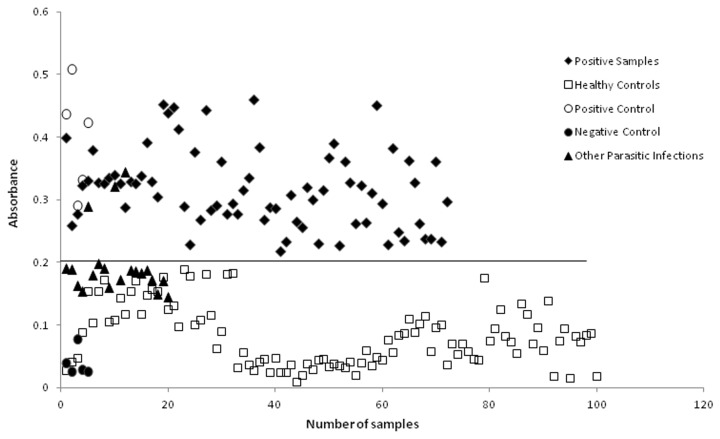
IgG ELISA absorbance (O.D) values. Serum samples obtained from 100 healthy blood donors (□), 72 positive samples from surveyed population (♦), five positive controls (○), five negative controls (●) and 20 samples from patients with other parasitic diseases (▲).

The cut-off OD was determined as mean absorbance ± 2SD of the value of 100 apparently healthy blood donors (Cut-off was 0.08 ± 0.05x2 = 0.20). Based on this cut off, out of 1429 samples, 72 (5.03%) were anti-*Echinococcus* IgG ELISA positive. Five surgically confirmed patients who served as positive controls were positive for anti-*Echinococcus* IgG antibodies by ELISA and five healthy controls which were negative for intestinal parasitic infections were negative for anti-*Echinococcus* IgG antibodies by ELISA, in each plate. Out of 20 samples from patients with other parasitic infections, 2 out of 5 samples from ascariasis patients and one out of 5 samples from amoebiasis patients were cross reactive in ELISA (Fig 2).

Univariate statistical analysis was conducted in relation to age, gender, residence and contact with dogs (Table 2). The results of the study show that age group of <15 years had significantly higher number of seropositve subjects (RR = 3.643, 95%CI = 2.052–6.467, P<0.001). Males were significantly more positive than females (RR = 1.831, 95%CI = 1.132–2.961, P = 0.012). Rural residence was found to be at a significantly high risk with a P value of (RR = 8.533, 95%CI = 2.079–35.020, P<0.001). Finally, contact with dogs was significantly a higher risk factor (RR = 3.11, 95%CI = 1.583–6.137, P<0.001).

**Table 2:** Association of potential exposure variables with seroprevalence of hydatid infection as determined by univariate analysis.

Risk Factors	N (Seropositive/total) (%)	RR (95% of CI)	P-value*
**Gender**			
*Male*	42/630 (6.6)	1.831(1.132–2.961)	0.012
*Female*	30/799 (3.75)		
**Residence**			
*Rural*	70/1161(5.26)	8.533(2.079–35.020)	<0.001
*Urban*	02/268 (4.59)		
**Age group**			
*1–15 years*	19/148 (5.79)	Reference	
*16–55 years*	41/1015 (4.07)	3.643 (2.052–6.467)	<0.001
*Above 55 Years*	12/266 (3.05)	3.118 (1.468–6.621)	0.002
**Contact with Dog**			
*Contact*	62/965 (6.42)	3.11 (1.583–6.137)	<0.001
*No contact*	10/464 (2.15)		

Binary logistic regression (Multivariate) analysis was conducted (Table 3) which indicated that subjects in the age group <15 years are having 3.18 times more chances of infection than 16–55 years (OR = 0.314, 95%CI = 0.175–0.565, P<0.001) and > 55 years of age (OR = 0.355, 95%CI = 0.165–0.764, P = 0.008). Males were having 1.83 times more chances of infection as compared to females (OR = 0.544, 95%CI = 0.333–0.888, P = 0.015) and subjects having contact with dogs are having 2.95 times more chances of infection as compared to subjects with no contact (OR = 0.338, 95%CI = 0.171–0.670, P = 0.002). Subjects living in rural areas were having 7.68 times more chances of infection as compared to the subjects living in urban areas (OR = 7.680, 95%CI = 1.861–31.695, P = 0.005).

**Table 3:** Binary logistic regression (Multivariate) analysis of the relationship of exposure variables and seroprevalence of hydatid infection, among the studied population of Kashmir Valley.

Variable	P-Value	OR (95% of CI)
**Age**		
*16–55 Years*	0.001	0.314(0.175–0.565)
*Above 55 Years*	0.008	0.355(0.165–0.764)
**Gender**		
*Female*	0.015	0.544(0.333–0.888)
**Contact with Dog**		
*No Contact*	0.002	0.338(0.171–0.670)
**Residence**		
*Rural*	0.005	7.680(1.861–31.695)

### Western blot analysis

All the 72 seropositive samples were reactive in Western blotting. IgG reactivity revealed multiple immunoreactive bands ranging from 28–134 kDa. The percentage occurrence of the highly immunoreactive antigenic fraction was 57 kDa (72.2%) followed by 70 kDa (66.7%) and 39kDa (58.3%) (Table 4). Sera from patients with other parasitic infections showed cross-reactivity with the cluster of 54–59 kDa bands. None of the samples from normal healthy individuals were immunoreactive.

**Table 4:** Number of serum samples reactive and percentage occurrence by Western blot assay (N = 72).

S.No	Reactive antigenic fraction (Mol wt in kDa)	Number of reactive samples (Occurrence)	Percentage Occurrence (immunereactivity)
1	28	4	5.6
2	39	42	58.3
3	52	2	2.8
4	57	52	72.2
5	60	12	16.7
6	63	4	5.6
7	70	48	66.7
8	76	8	11.1
9	94	12	16.7
10	110	6	8.3
11	123	4	5.6
12	134	10	13.9

## Discussion

Cystic echinococcosis (CE) has a worldwide geographical distribution. It is found in all continents, with the highest prevalence in parts of the Russian Federation and adjacent independent states, China, north and east Africa, Australia and South America. In Europe; it is present in every country or region. The annual incidence of human CE varies between <1 and >8 per 10^5^ population with the exception of Ireland, Iceland and Denmark. It is most intensively endemic in parts of Spain, southern Italy, Sardinia, and in India. Reports from several countries also provide documented evidence of an increase in the CE incidences in recent years [21, 22]. In India the annual incidence varies from 1–200/100,000 population. High prevalence is reported from Kashmir, Andhra Pradesh, Tamil Nadu and Central India [23].

ELISA was used in the present study, which is highly sensitive and specific technique for detection of hydatid specific antibody. A study by Kaur *et al*., reported a sensitivity of 100% and a specificity of 90.27% for the standard ELISA as compared to rapid ELISA (82.3%) and IHA (70.58%) [24]. Chirag *et al*., reported a sensitivity of 95.12% and specificity of 87.5% using crude antigen [16]. Although ultrasonography is essential to reveal cases which should be benefited by treatment, serology may provide useful data for assessing the actual infection. Moreover, an ultrasound examination is not helpful in most cases having extrahepatic infections.

In the present study, the seropositivity of *Echinococcus* infection in an asymptomatic population was found to be 5.03% by ELISA. Further higher number of seropositive subjects were found in Anantnag district (9.23%) followed by Baramulla (8.5%) and Bandipora (8.0%) districts. In a study from China a high seroprevalence of 25.5% was reported by the indirect hemagglutination assay (IHA) in a Tibetian population. The high prevalence reported in the above study may be because this study was conducted in nomadic population, who are at high risk of having an infection [25]. In a retrospective analysis of clinically suspected cases of hydatidosis from North India 15% and 37.21% seropositivity has been reported [3,26]. High prevalence reported in these studies may be because only clinically suspected patients were included in the study and moreover hospital based studies are not the true indicators of prevalence in the community. In an epidemiological study carried out in Iran, China and Central Greece a low prevalence ranging from 1–3% was observed [27–29]. A prevalence of 3.46% was observed in central Iran and Spain [30–31].

Immunoblotting results of 72 ELISA seropositive samples in the present study identified 12 major discrete immunoreactive antigenic fractions ranging from (28–134 KDa) molecular mass, with the use of hydatid cyst fluid (HCF) obtained from sheep. These findings are in agreement with those obtained by Kanwar *et al* [32]. In the present study, the highly immunoreactive antigenic fraction was 57 kDa (72.2%) followed by 70 kDa (66.7%) and 39kDa (58.3%). Sera from patients with other parasitic infections showed cross-reactivity with the cluster of 54–59 kDa bands.

High seropositivity in the asymptomatic subjects in the present study can be explained by the presence of huge population of stray dog in the region. While WHO-sponsored national multi-centric survey puts the average human-dog ratio in the country at 1:36 [33] the ratio in the Kashmir Valley stands at 1:12. High population of stray dogs who invariably wanders on main roads particularly near butcher shops may be a source of dissemination of infection. The slaughtering of livestock without veterinary control, the widespread rural practice of feeding dogs with the viscera of home butchered sheep and the lack of public health education are the main reasons for the high prevalence [34]. During the season from spring to autumn nomadic population move in search of pastures with their herd of sheep and they always keep sheepdog to protect the herd from wild animals. They live in temporary houses with unhygienic condition and dog rending in predomestic area. All these factors are favorable for transmission of hydatidosis.

A major risk factor in the study was the age group up to 15 years significantly associated with the seropositivity. The higher number of seropositive asymptomatic subjects in younger population suggests ongoing transmission in the region which could be responsible for recent increase in infection in younger age group. High prevalence in children might be because of the exposure to the contaminated environment. Stray dogs mostly occupy open areas like parks and playground which are preferred sites for children to play thus are exposed to parasite eggs. Parasite eggs survive and remain infective for months under favorable conditions such as high humidity and low temperature [35]. Seropositivity in the young age group may or may not be associated with the further development of hydatid cyst, as an initial exposure may lead to progressive lesion, abortive lesion or spontaneous cure. Infection due to *Echinococcus* in the initial phase for many years is always asymptomatic. The infected subjects may or may not be symptomatic later in life depending upon the host immunity and other associated factors. The infection may become symptomatic if the cysts either rupture or exert a mass effect [36]. Survey in children is a useful tool to monitor changes of transmission dynamics in humans, and to provide ‘warning signals’ to decision makers for the institution of specific control measures. The annual incidence of CE in children increased from 0.7 per 10^5^ in 1971–1982 to 5.4 in 1995 in Bulgaria [37]. Another study has reported 10–19 years of age as highest infected age group in Zanjang [38]. The data indicate that most human infections with *E*. *granulosus* occur during childhood and adolescence. In evaluating the epidemiology of echinococcosis or the effectiveness of a control programme, therefore, reductions or increases in the incidence of clinical disease among children and adolescents indicate an improving or worsening situation, respectively [39]. In contrast, in other studies the increased seroprevalence was seen in the elderly population. Higher prevalence in adults as compared to children’s suggests reduction in disease burden, due to the control measures taken [29]. There are contrasting reports of seropositivity in different age groups and most of the reports are based on hospital studies, which mainly include symptomatic subjects. The prepatent period is very long in this disease and most cases present with symptoms years after infection so it is difficult to detect true age group when infection has occurred.

Seropositivity in males in the present study was significantly higher than in females. This finding could be explained by the culture of the area, where men are more involved in farming and herding livestock and more likely than women to come in contact with dogs. A similar observation was made by Heidari *et al* [27]. Our finding is in sharp contrast with that of Yang *et al* & Craig *et al*., who reported echinococcosis predominantly in females [40, 41].

Another risk factor identified in the present study was the rural residence. High prevalence in rural areas may be because the population is closely related to *Echinococcus* biological cycle. Moreover, poor economic conditions, low education levels and poor medical services in rural people are other factors responsible for high prevalence. Contamination of soil by dog feces is a common occurrence. Farming is the main occupation in rural areas thus rural population is at a high risk of acquiring infection because people come in contact with contaminated soil and inhale dust containing eggs during farming activity [35].

Similarly, contact with dog was significantly associated with seropositivity. Most forms of human CE transmitted in domestic life cycles involving dogs and livestock. The most known is the sheepdog cycle. In recent years, it has been recognized by genetic characterization that in the genus of *Echinococcus* there are distinct species and strains (e.g. sheep, pig, cattle, cervid, and camel strain) based on morphology, host specificity and molecular characteristics. Previously we have demonstrated the presence of G3 (Buffalo strain) as predominant genotype followed by G1 (Sheep strain) and G2 (Tasmanian Sheep strain) genotype of *E*.*granulosus* in ungulate animals in North India. The genetic diversity and population genetics of *E*.*granulosus* senso stricto complex analyzed based on sequencing of mitochondrial DNA suggests high genetic diversity within the population of *E*.*granulosus* and relatively low to high level of genetic differentiation among the populations. Genotyping of human cases of CE play an important role in the formulation of control strategies for the prevention of transmission of this parasite [42, 43].

Higher number of seropositive asymptomatic subjects suggests active transmission of infection thus supporting unhygienic practices and habits being adopted. People in rural areas need to be educated about proper control measures, since infection occurs through accidental ingestion of *Echinococcus* eggs. Also, feeding of livestock remains to dogs should be avoided as it is a starting point for *E*.*granulosus* life cycle. The study suggests that education program for echinococcosis control should be initiated in school children because most infections acquire during childhood. The transmission of the infection can be reduced by proper slaughtering, safe waste disposal of infected animals, incineration of infected viscera, provision of safe drinking water, controlling of the dog population and their antihelminthic treatment. Proper education creating awareness and implementation of strict rules regarding disposal of remains of slaughtered animals can help to eradicate this disease.

The limitations of the present study are that the number of subjects enrolled in the age group <15 year are less than in higher age groups. Moreover, the other parasitic infections cannot be ruled out in the study subjects (1429), which may have shown non-specific ELISA seropositivity. By Western blotting, samples from patients with other parasitic infections showed cross reactivity with the cluster of 54–59kDa molecular mass fractions. The immunoreactivity of 52 (72.2%) out of 72 samples with 57 kDa antigenic fraction may be due to cross reactive antigens with other parasitic infections, especially ascariasis prevalent in the region. However, out of 72 ELISA seropositive samples, 48 (66.7%) and 42(58.3%) samples were immunoreactive with 70 and 39 kDa specific antigenic fractions respectively, supporting the ELISA results to a great extent and indicating the possibility of asymptomatic infection in the population.

## Conclusion

The study revealed the ongoing transmission and asymptomatic infection in most of the areas in the study region and indicates that CE is a public health problem in Kashmir, North India, There is an urgent need for increasing the public awareness about the disease and to monitor the prevalence in exposed populations. Moreover, there is no previously published study from this region regarding prevalence of hydatid infection in an asymptomatic population; hence the present study will serve as baseline data for monitoring future changing trends of this infection and thus may help in the formulation of control strategies.
